# Baseline neuronal antibodies in patients with small cell lung cancer are not necessarily associated with post-immune checkpoint inhibitors neurotoxicities

**DOI:** 10.3389/fimmu.2025.1681765

**Published:** 2025-11-20

**Authors:** Simone Rossi, Elisa Andrini, Rita Rinaldi, Tania Silvestri, Maria Giovanna Formelli, Adriana Di Odoardo, Barbara Lenzi, Maria Guarino, Davide Campana, Giuseppe Lamberti

**Affiliations:** 1 IRCCS Istituto Delle Scienze Neurologiche di Bologna, Bologna, Italy; 2 Dipartimento di Scienze Mediche e Chirurgiche, Università degli Studi di Bologna, Bologna, Italy; 3 Metropolitan Laboratory, AUSL Bologna, Bologna, Italy; 4 Dipartimento Malattie oncologiche ed ematologiche, IRCCS Azienda Ospedaliero-Universitaria di Bologna Policlinico di Sant’Orsola, Bologna, Italy

**Keywords:** immunotherapy, immune checkpoint inhibitor, small cell lung cancer (SCLC), neurotoxicity, paraneoplastic neurologic syndrome

## Abstract

**Background and objectives:**

Autoantibodies against intracellular neuronal antigens (IC-Abs) can be found in neurologically asymptomatic patients with small cell lung cancer (SCLC) and have been proposed as a predictive biomarker for the development of post-immune checkpoint inhibitors (ICIs) neurotoxicities. The aim of this study was to prospectively evaluate the association of baseline neural antibodies with immune-related adverse events (irAEs) - including neurological irAEs (n-irAEs) - and oncological outcomes in patients with SCLC following ICI therapy.

**Methods:**

In this prospective cohort study, consecutive patients with SCLC eligible for treatment with ICI were assessed for the presence of IC-Abs with both indirect immunofluorescence (IIF) tissue-based assay (TBA) and line-blot and underwent baseline neurological evaluation prior to ICI initiation. Patients were longitudinally monitored for irAEs occurrence and oncological outcomes. Comparisons between groups, time-to-event and multivariable analyses were performed.

**Results:**

Fifty-six neurologically asymptomatic patients with SCLC (median age 70.5 years, 38% female) were included. Nineteen (34%) had IC-Abs prior to ICI-treatment (anti-Hu, n=7 [37%]; anti-Zic4, n=6 [32%]; anti-SOX1, n=3 [16%]; anti-SOX1 and anti-Zic4, n=2 [11%]; anti-Purkinje cerebellar cells, n=1 [5%]). Following ICI-treatment, two patients (3.6%) developed a n-irAE (one with baseline anti-Hu antibodies; one without baseline IC-Abs). The presence of baseline IC-Abs was not associated with an increased incidence of n-irAEs. However, anti-Hu antibody positivity was associated with an increased risk of irAEs of any type (OR 8.3; 95% CI, 1.22–56.54). A non-significant trend toward longer progression-free survival was observed in anti-Hu–positive patients (9.4 vs 5.7 months; p=0.10).

**Discussion:**

The presence of baseline IC-Abs may not be associated with the occurrence of post-ICI neurotoxicities in patients with SCLC. However, anti-Hu antibody positivity correlates with an increased risk of irAEs of any type. Larger studies are needed to assess the safety of ICI therapy in patients with SCLC harbouring neural antibodies and to investigate their potential role as predictive biomarkers of post-ICI neurotoxicities.

## Introduction

Immune checkpoint inhibitors (ICIs) have revolutionized the therapeutic landscape of several malignancies, including small-cell lung cancer (SCLC), and programmed death-ligand 1 (PD-L1) inhibition (with either atezolizumab or durvalumab) - added to etoposide and either carboplatin or cisplatin chemotherapy - has become the standard first-line treatment for extensive-stage (ES)-SCLC (1, 2). Despite their clinical efficacy, ICIs can activate the immune system in a non-specific manner, potentially leading to off-target autoimmune adverse events – collectively termed immune-related adverse events (ir-AEs) –, which can affect any tissue or organ system, including the nervous system (3). Neurological ir-AEs (n-irAEs) occur in 1-3% of patients treated with ICIs and encompass a broad clinical spectrum, including myositis, myasthenic syndromes, neuropathies and encephalitis (4). Remarkably, a subset of n-irAEs closely resembles spontaneous paraneoplastic neurological syndromes (PNSs) (5), a group of autoimmune neurological disorders triggered by a remote cancer – most commonly SCLC – and typically associated with autoantibodies against intracellular neuronal antigens (IC-Abs) ectopically expressed by cancer cells (6, 7). Retrospective data suggest that ICI treatment may unmask a latent paraneoplastic autoimmunity, as evidenced by an increased incidence of anti-Ma2 and anti-Hu associated PNSs following widespread ICI usage (8, 9). Moreover, IC-Abs have been detected in samples, collected before ICI initiation, from patients who developed a PNS-like n-irAE (10–12). Consequently, IC-Abs have been proposed as potential predictive biomarkers for the development of ICI-induced PNSs, particularly after the approval of PD-L1 inhibitors in SCLC, a tumor inherently prone to boost paraneoplastic autoimmunity. Nevertheless, prospective data on safety of ICI treatment in SCLC patients with pre-treatment neural antibodies are lacking. Furthermore, the impact of baseline neural antibodies on survival outcomes in ICI-treated SCLC patients remains poorly explored.

In this study, we sought to characterize the prevalence of baseline IC-Abs in ICI-naïve patients with SCLC and to assess their impact on the occurrence of neurological and non-neurological adverse events and survival outcomes.

## Materials and methods

### Study design and patient selection

In this monocentric prospective cohort study, we enrolled all consecutive patients with ES-SCLC – including those with ES at diagnosis and those who progressed to from limited to extensive stage during the treatment – who were eligible to receive chemoimmunotherapy with an ICI according to standard clinical practice between January 1^st^, 2021, and December 31^st^, 2024, and who provided informed consent to participate in the study. Patients with other high-grade neuroendocrine tumors of the lung, i.e., large-cell neuroendocrine carcinoma (LCNEC) or combined SCLC-LCNEC, who received ICI-based treatment were excluded from the study.

The following baseline characteristics were collected: sex, age at ICI start, performance status according to Eastern Cooperative Oncology Group (ECOG PS), pathology diagnosis and Ki-67 value, disease stage at ICI start, and type of ICI received. Patients were prospectively monitored for oncological outcomes, and we collected data on treatment start and end date, best radiological response, date of radiological progression (or last radiological evaluation) and survival status (or last contact).

Prior to ICI initiation, all patients underwent a comprehensive neurological evaluation (S.R., R.R.) and were tested for baseline serum antibodies against intracellular neuronal antigens. Before performing the initial neurological assessment, the neurologists who conducted the examination were blinded to the results of the immunological assay. Patients with clinically evident PNSs were considered ineligible for ICI treatment and were therefore excluded from the subsequent analysis. After ICI initiation, neurological assessment was performed every 3 months until patient refusal, study completion, or death.

The diagnosis of n-irAEs was based on the temporal relationship between neurological symptoms onset and ICI administration (i.e., within 12 months of the last ICI infusion) and the accurate exclusion of other potential etiologies (e.g., cancer dissemination, central nervous system infections, other chemotherapy-induced toxicities) through a comprehensive diagnostic work-up, as recommended by the consensus definition paper (13). In patients with n-irAEs, we recorded ancillary diagnostic data (including CSF analysis, nerve conduction studies, electroencephalogram, and brain MRI imaging), as well as details on the management of the n-irAE.

The search for antibodies against intracellular neuronal antigens was conducted in the time interval between SCLC diagnosis and ICI initiation and was performed using 2 techniques, as recommended elsewhere (14, 15). Samples were tested for reactivity using a commercial indirect immunofluorescence (IIF) tissue-based assay (TBA) on monkey-derived sections of cerebellum and intestinal tissue (Euroimmun, Lubeck, Germany), followed by a confirmatory line-blot assay, which included anti-Hu, anti-Yo, anti-Ri, anti-Ma2, anti-amphiphisin, anti-CV2/CRMP5, anti-recoverin, anti-SOX1, anti-titin, anti-Zic4, anti-GAD65, and anti-Tr/DNER (Euroimmun, Lubeck, Germany). Antibody titers were derived from line-blot band intensity, using a semiquantitative scoring system (0 = negative, 1 = weak positive, 2 = positive, 3 = strong positive). Isolated line-blot positivity without corresponding IIF-TBA staining was considered non-significative.

### Statistical analysis

Categorical and continuous variable were compared using Chi-squared, Fisher’s exact test, or Mann-Whitney U-test, as appropriate. Survival outcomes were estimated with the Kaplan-Meier method and compared with the log-rank method. Overall survival (OS) was defined as the time between the start of the treatment with ICI and death from any cause. Progression-free survival (PFS) was defined as the time from the start of the treatment with ICI and radiological progression or death, whichever occurred first. Patients alive and progression-free at the time of analysis were censored at the time of last radiological assessment without evidence of disease progression. Patients still alive at the time of analysis were censored at the time of last contact. Tumor response was assessed according to standard RECIST v1.1 criteria. Both neurological and non-neurological ir-AEs were recorded at each visit, as per standard practice. The severity of irAEs and n-irAEs was assessed using the Common Terminology Criteria for Adverse Events (CTCAE) version 5.0. Multivariable analysis for the risk of development of irAE and n-irAE was conducted with a generalized linear model and estimated with ratio of the odds (OR). All estimates were reported with 95% confidence interval (95%CI). Statistical analyses were conducted with R software v. 4.2.2.

### Ethics statement

The study protocol was approved by the local ethic committee (approval number 53/2021/SPER/AUSLBO) and was conducted in accordance with the principles of the Declaration of Helsinki (6th revision, 2008). All patients or their legal representatives provided written informed consent for all the procedures related to the study.

### Data availability

Anonymized data will be shared to any qualified researcher upon reasonable request.

## Results

### Study population

Among 136 patients with a diagnosis of high-grade neuroendocrine carcinoma of the lung between January 1^st^ 2021 and December 31^st^ 2024, a total of 58 patients with ES-SCLC (49 at the diagnosis, 9 with limited-stage disease at the diagnosis that progressed to ES-SCLC) were eligible to receive chemoimmunotherapy and were enrolled in the study. Two patients presented a clinically manifest paraneoplastic neurological syndrome that, in both cases, predated the diagnosis of cancer (one patient with limbic encephalitis and anti-Hu antibodies; one patient with basal ganglia encephalitis and sensory neuropathy and anti-Hu antibodies). These two patients were deemed ineligible to ICI treatment and were not considered in the subsequent analysis.

Thus, 56 patients received chemoimmunotherapy and were included in the analysis (**Supplementary Figure 1**). The median age was 70.5 years [range 51-85] and 38% were female. The median time from SCLC diagnosis and ICI initiation was 2.5 weeks (range: 2-4).

Nineteen patients (34%) had baseline IC-Abs prior to ICI initiation, including anti-Hu (n=7, 37%), anti-Zic4 (n=6, 32%), anti-SOX1 (n=3, 16%), and combined anti-SOX1 and anti-Zic4 (n=2, 11%). The median line-blot band intensity obtained by line-blots analyses in antibody-positive patients was 2 (range: 1-3). One patient (5%) showed positive IIF-TBA staining targeting Purkinje cerebellar cells, without identifiable specificity at line-blot assay. Three patients exhibited isolated line-blot positivity for SOX-1 antibodies – two with ‘positive’ reactivity (2/3) and one with ‘weak positive’ reactivity (1/3 line-blot band intensity) – but tested negative on IIF-TBA and were therefore considered as antibody-negative.

Two patients (both harbouring baseline IC-Abs) had a history of autoimmune diseases (one patient with baseline anti-Hu antibodies had a history of systemic erythematous lupus with cutaneous and articular involvement; one patient with anti-SOX1 and anti-Zic4 antibodies had multiple sclerosis in clinical remission and without specific immune-active treatment at the time of study enrolment). Baseline demographic and clinical characteristics were comparable between antibody-positive and antibody-negative patients, except for a trend toward younger age at diagnosis in the antibody-positive group (68 vs 73 years; p=0.053). Patient characteristics of the overall cohort and comparison between patients with and without neural antibodies are summarized in **Table 1**, while **Supplementary Table 1** reports the comparison between patients with and without baseline anti-Hu antibodies.

**Table 1:** Patient characteristics in the study population and by neural antibody positivity.

	Overall (N = 56)	Neural Abs Negative (N = 37)	Neural Abs Positive* (N = 19)	p-value
Sex				
Female	21 (37.5%)	11 (29.7%)	10 (52.6%)	0.145
Male	35 (62.5%)	26 (70.3%)	9 (47.4%)	
Age				
Years, median [range]	70.5 [51 – 85]	73 [51 – 84]	68 [55 – 85]	0.053
Pack-year				
Median [range]	50 [0 – 100]	60 [0 – 80]	46 [5 – 100]	0.238
ECOG PS				
0	17 (30.4%)	11 (29.7%)	6 (31.6%)	1.00
≥1	39 (69.6%)	26 (70.3%)	13 (68.4%)	
Stage at diagnosis				
Extensive	47 (83.9%)	30 (81.1%)	17 (89.5%)	0.703
Limited	9 (16.1%)	7 (18.9%)	2 (10.5%)	
Ki-67				
%, median [range]	80 [50 – 95]	85 [50 – 90]	80 [70 – 95]	0.455
Metastatic sites before ICI start				
Liver	15 (26.8%)	11 (29.7%)	4 (21.1%)	0.543
Bone	13 (23.2%)	9 (24.3%)	4 (21.1%)	1.00
Brain	9 (16.1%)	5 (13.5%)	4 (21.1%)	0.470
Type of ICI				
Atezolizumab	38 (67.9%)	27 (73.0%)	11 (57.9%)	0.363
Durvalumab	16 (28.6%)	9 (24.3%)	7 (36.8%)	
Pembrolizumab	2 (3.6%)	1 (2.7%)	1 (5.3%)	
ICI cycles				
Median [range]	7 [1 – 21]	7 [1 – 18]	7 [2 – 21]	0.945

*Includes positive testing for any of the following: anti-Hu, anti-Yo, anti-Ri, anti-Ma2, anti-amphiphisin, anti-CV2/CRMP5, anti-recoverin, anti-SOX1, anti-titin, anti-Zic4, anti-GAD65, and anti-Tr/DNER.

p-values from Fisher’s exact test, Chi-squared, or Mann-Whitney U test as appropriate.

ECOG PS, Eastern Cooperative Oncology Group performance status; ICI, immune checkpoint inhibitor.

### Neurological immune-related adverse events

**Figure 1:**
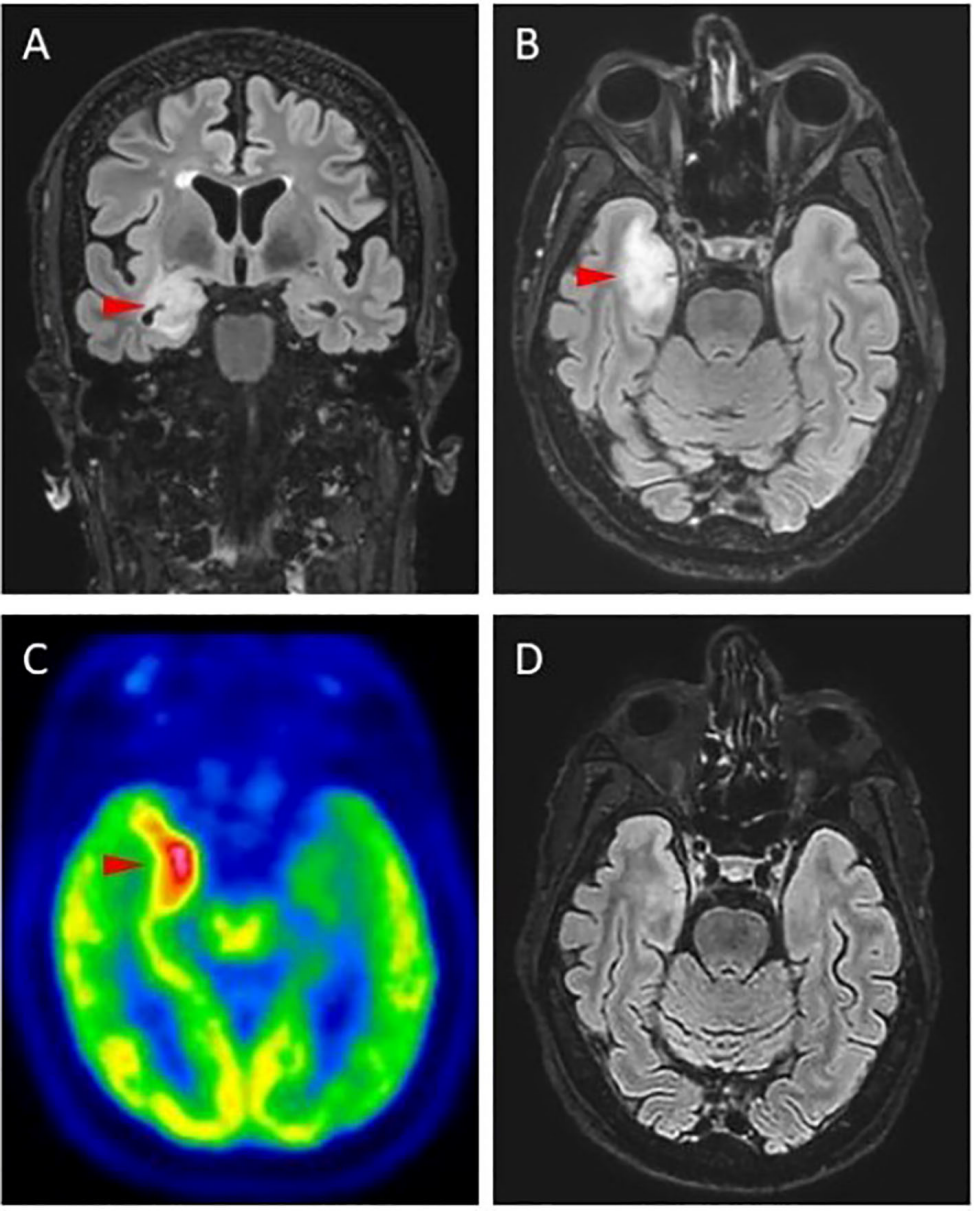
Brain MRI of a patient who developed limbic encephalitis after ICI treatment. **(A, B)** FLAIR sequences showing hyperintensity and swollen appearance of the right mesial temporal lobe in **(A)** coronal and **(B)** axial view. **(C)** 18-Fluoro-deoxyglugose positron emission tomography (18F-FDG-PET) showing an area of intense tracer uptake corresponding with MRI abnormalities. **(D)** Control brain MRI performed two weeks after pulse steroids (1 g/die for 5 days) showed complete resolution of the abnormalities.

**Figure 2:**
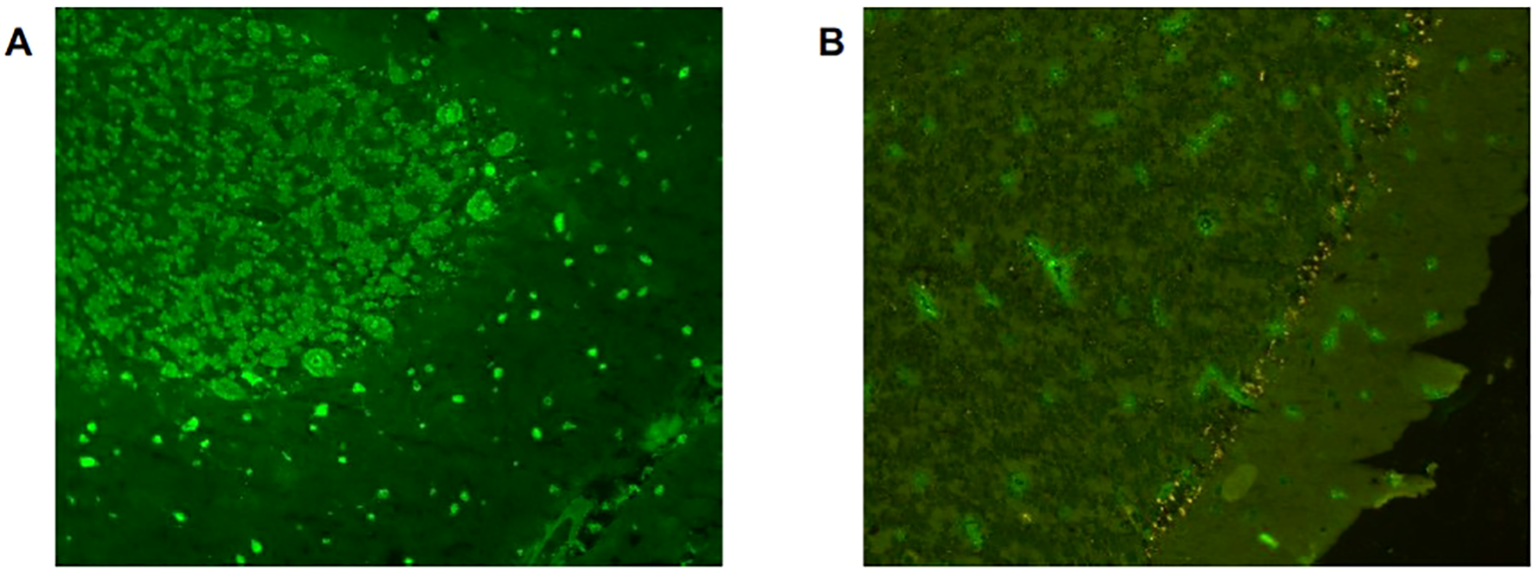
Indirect immunofluorescent assay on rat brain section of a patient with limbic encephalitis. **(A)** Pre-treatment serum shows strong nuclear staining in neurons of the hippocampus, consistent with anti-Hu antibody positivity. **(B)** Following the onset of immune-related encephalitis, the serum from the same patient shows absence of neuronal nuclear reactivity. Immunostaining was performed with serum at 1:200 dilution and visualized using FITC-conjugated anti-human IgG (green).

Two patients (3.6%) developed a neurological toxicity following ICI treatment. One patient, who tested negative for baseline neural antibodies, developed a subacute demyelinating polyradiculoneuropathy after a single ICI infusion, that was successfully treated with oral prednisone (1 mg/kg/day). The other patient, who harboured baseline high-titer anti-Hu antibodies (“strong positive” at line-blot analyses), developed a limbic encephalitis (**Figures 1A-C**) following 12 cycles of atezolizumab, while he was concomitantly exhibiting a significant tumor reduction Surprisingly, when he was retested after the onset of the neurotoxicity, the search for neural antibodies (including both IC-Abs and antibodies against cell-surface neuronal antigens) was negative in both serum and CSF (**Figures 2A, B**). The patient was treated with pulse methylprednisolone, followed by slowly tapering, with significant clinical and radiological response (**Figure 1D**). Despite the neurological improvement, the patient was not rechallenged with ICI and died of cancer progression eight months after the onset of the neurotoxicity. The search for serum IC-Abs was not repeated following the resolution of ICI-encephalitis and the tumor progression.

Overall, the incidence of n-irAEs did not differ significantly between antibody-positive and -negative patients (1/19 [5.2%] vs 1/36 [2.7%], p=0.62).

### Immune-related adverse events of any type

A total of 14 patients (25%) developed at least one irAE during ICI treatment. In addition to the two patients with n-irAEs, 12 patients experienced non-neurological irAEs, including hyperthyroidism (n=6; four grade 1, two grade 2), pneumonitis (n=2, grade 2), hypothyroidism (n=1, grade 1), pancreatitis (n=1, grade 3), hepatitis (n=1, grade 3), and asymptomatic lipase elevation (n=1, grade 3). Detailed comparisons between patients with and without ir-AEs are presented in **Table 2**.

**Table 2:** Characteristics of patients by occurrence of immune-related adverse events (irAE).

	No irAE (N = 42)	irAE (N = 14)	p-value
Sex			
Female	14 (33.3%)	7 (50.0%)	0.343
Male	28 (66.7%)	7 (50.0%)	
Age			
Years, median [range]	71.5 [51 - 85]	69 [55 - 82]	0.358
Pack-year			
Median [range]	51 [0 - 100]	50 [20 - 90]	0.373
ECOG PS			
0	12 (28.6%)	5 (35.7%)	0.739
≥1	30 (71.4%)	9 (64.3%)	
Stage at diagnosis			
Extensive	36 (85.7%)	11 (78.6%)	0.676
Limited	6 (14.3%)	3 (21.4%)	
Ki-67			
%, median [range]	80 [50 - 95]	85 [70 - 90]	1.00
Metastatic sites at ICI start			
Liver	14 (33.3%)	1 (7.1%)	0.082
Bone	12 (28.6%)	1 (7.1%)	0.149
Brain	9 (21.4%)	0 (0%)	0.093
Type of ICI			
Atezolizumab	29 (69.0%)	9 (64.3%)	0.613
Durvalumab	12 (28.6%)	4 (28.6%)	
Pembrolizumab	1 (2.4%)	1 (7.1%)	
ICI cycles			
Median [range]	6.5 [1 - 15]	10.5 [4 - 21]	0.003
Neural antibodies			
Negative	29 (69.0%)	8 (57.1%)	0.518
Positive	13 (31.0%)	6 (42.9%)	
Anti-Hu antibodies			
Negative	40 (95.2%)	9 (64.3%)	0.008
Positive	2 (4.8%)	5 (35.7%)	

ECOG PS, Eastern Cooperative Oncology Group performance status; ICI, immune checkpoint inhibitor.

While the overall neural antibody positivity did not differ between patients who developed at least one ir-AE and those who did not (43% and 31% respectively; p=0.518), the frequency of anti-Hu positivity was significantly higher in patients who developed at least one irAE (36% vs 4.8%; p=0.008). Accordingly, irAEs incidence was significantly higher among anti-Hu–positive patients (5/7; 71%) compared to anti-Hu–negative patients (9/49; 18%) (p=0.008). After adjusting for treatment duration, baseline anti-Hu antibody positivity remained significantly associated with irAE development (adjusted OR 8.3; 95% CI: 1.22–56.54; p=0.03). Nevertheless, the severity of ir-AEs was not associated with the presence of IC-Abs or anti-Hu antibodies. Four grade 3 irAEs were observed: two in the IC-Abs–positive group (one encephalitis and one asymptomatic lipase increase, both anti-Hu–positive) and two in the IC-Abs–negative group (one hepatitis and one pancreatitis). Fisher’s exact tests showed no significant association between IC-Abs or anti-Hu antibody positivity and the occurrence of grade 3 *versus* lower-grade irAEs or no irAEs (p = 1.00 and p = 0.58 for IC-Abs, and p = 0.59 and p = 0.072 for anti-Hu, respectively; **Supplementary Table 2**). Lastly, as previous studies have correlated other autoantibodies to the occurrence of irAEs (16, 17), we analyzed the association of those routinely performed at our Institution as per clinical practice, which included anti-nuclear antibodies (ANA), anti-thyroid stimulating hormone receptor (TSH), anti-thyroid peroxidase (TPO), anti-thyroglobulin (Tg), and anti-neutrophil cytoplasmic antibody (ANCA; i.e., proteinase 3 [PR3] and myeloperoxidase [MPO]). Antibodies against extractable nuclear antigens (ENA), including anti-Sm, anti-RNP, anti-SS-A (Ro), and anti-SS-B (La), anti-Jo1, and anti Scl-70, are performed on a “reflex” basis. We observed that antithyroid positivity and anti-ENA positivity were significantly associated with the development of irAEs (**Supplementary Figure 3**). Thus, we fitted a multivariable regression model to analyze the independent association of anti-Hu and each antibody positivity to irAE development. To avoid collinearity with ANA, only “ANA w/borderline” was included in the model, which had the lower p-value for the association with irAE occurrence compared to ANA. ENA antibodies were not included in the model because all cases with available ENA (14/56, 25%) did not have detectable anti-Hu antibodies. Anti-Hu were the only antibodies to show independent association with the development of irAEs with an odds ratio of 21.4 (p=0.004) (**Supplementary Figure 4**).

### Survival outcomes

At a median follow-up of 17.2 months (95%CI: 15.3 –NA), median PFS was 5.8 months (95%CI: 5.2 – 7.4) and median OS was 10.2 months (95%CI: 8.3 – 15.2) in the overall cohort.

Outcomes were similar among patients with and without baseline neuronal antibodies (PFS 5.7 vs 5.8 months, respectively [p=0.10]; OS 9.7 vs 10.2 months, respectively [p=0.9], **Supplementary Figure 1**).

**Figure 3:**
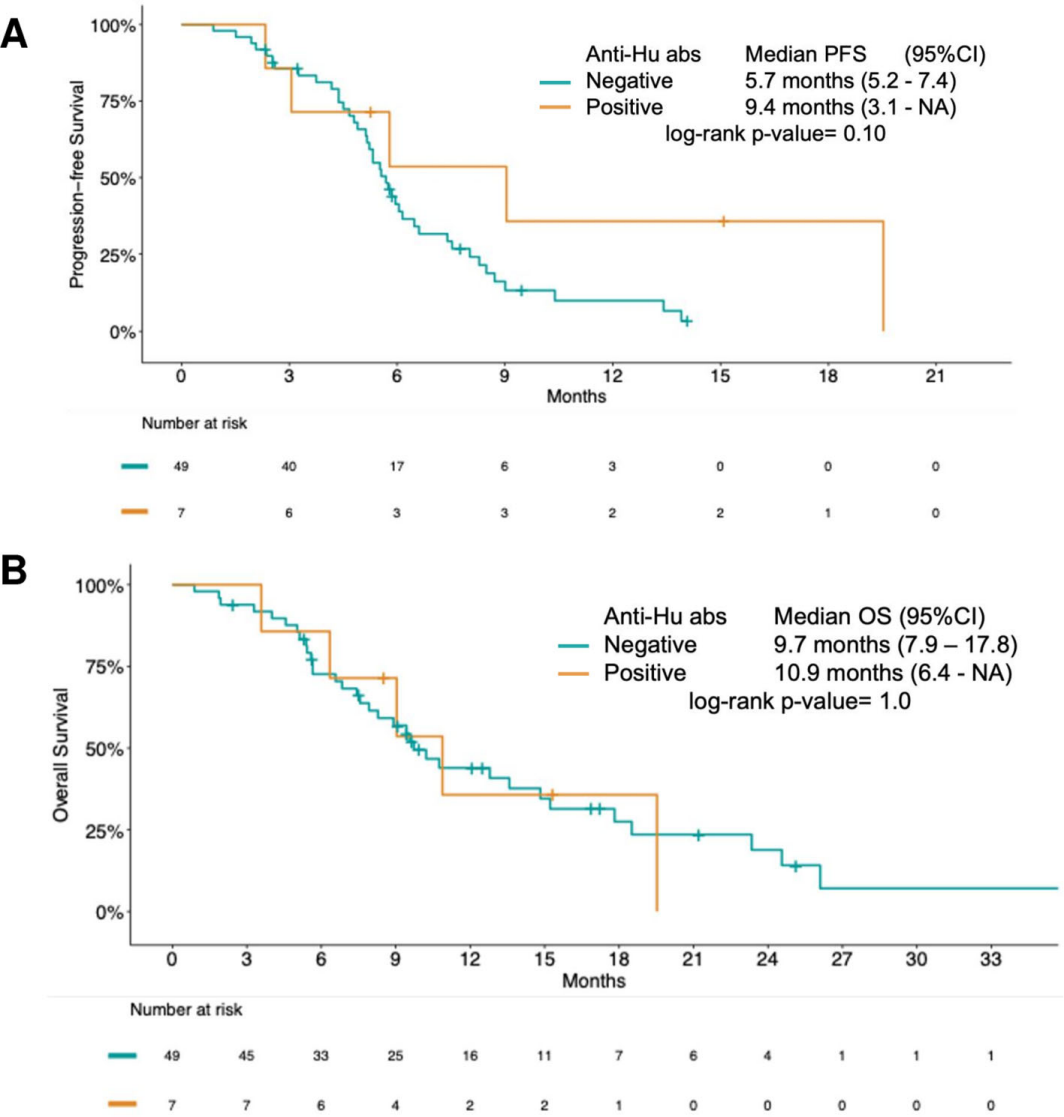
Treatment outcomes of study population by anti-Hu antibodies (abs) positivity. **(A)** Kaplan-Meier estimates of progression-free survival and **(B)** overall survival. 95%CI: 95% confidence interval.

Comparing patients with and without baseline anti-Hu antibodies, a trend toward a longer PFS was observed in anti-Hu–positive patients (9.4 vs 5.7 months; p=0.10), while OS (10.9 vs 9.7 months; p=1.0) was comparable between groups (**Figure 3**).

## Discussion

This study assessed the prevalence of baseline antibodies to intracellular neuronal antigens and prospectively evaluated their association with immune-related adverse events and oncological outcomes in patients with SCLC treated with ICIs.

Approximately one-third of patients harboured baseline neural antibodies, although only a minority exhibited neurological symptoms, highlighting that autoimmunisation against neural antigens is a necessary step, but is not sufficient for the development of clinically manifest PNS (18).

The presence of baseline neural antibodies was not associated with an increased risk of developing neurological toxicities following ICI treatment. However, patients with baseline anti-Hu antibodies presented a higher incidence of immune-related toxicities and a trend toward improved progression free survival.

From a pathophysiological standpoint, baseline neural antibodies in patients with SCLC may represent an epiphenomenon of a latent, tumor-driven immune response against neural antigens ectopically expressed by cancer cells. In this regard, the neuroendocrine origin of SCLC accounts for the frequent expression of neuronal antigens such as HuD (5, 6, 19), thereby explaining the high seroprevalence of these antibodies, as demonstrated in our and previous studies (5, 9). While this spontaneous immune activation is often insufficient to elicit an overt PNS (6), immune checkpoint (20)blockade may act as a “second hit” that amplifies the pre-existing immune response to a clinically apparent point (5). Supporting this hypothesis, neural antibodies have been retrospectively detected in serum samples collected prior to ICI initiation of patients with n-irAEs (8, 9, 11). Consequently, pre-treatment neural antibodies have been proposed as candidate biomarkers for ICI-induced PNS (5). However, to date, few studies have prospectively investigated the association between pre-treatment IC-Abs and ICI-induced toxicities. A recent study reported that non-neurological irAEs were more frequent in SCLC patients with pre-existing non-neural autoantibodies (rheumatoid factor, antinuclear antibodies or antithyroid antibodies), whereas no neurotoxicities were observed in the study cohort (17). Another prospective study found that three of 15 patients with pre-existing anti-SOX2 or anti-Hu antibodies developed severe post-ICI neurological toxicities, compared with none of the patients without those antibodies (16). However, both studies lacked comprehensive neurological assessments, and the identification of n-irAEs may have been under- or over-reported. Our study builds on the existing literature by providing comprehensive and sequential neurological evaluations, ensuring an accurate identification of neurological adverse events.

Our prospective findings suggest that the presence of baseline neural antibodies does not necessarily confer susceptibility to ICI-induced neurotoxicity. Notably, the case of ICI-associated encephalitis in our cohort raises additional mechanistic considerations. In this patient, anti-Hu antibodies – initially detected at a high titer at time of SCLC diagnosis – became undetectable following the onset of neurotoxicity. This observation challenges a straightforward neuroimmunological interpretation, particularly in light of previous reports showing that anti-Hu antibodies persist throughout chemotherapy in patients with SCLC (21). A plausible hypothesis is that the marked tumor shrinkage achieved with chemo-immunotherapy may have mitigated the cancer-driven immune response, leading to the disappearance of the ‘paraneoplastic’ antibodies (22). Concurrently, ICI exposure may have triggered a *de novo*, cancer-independent, immune response. The favourable response to corticosteroids further supports an immune-mediated mechanism distinct from classic anti-Hu-associated PNS, which typically exhibit poor responsiveness to immunosuppression (23).

Intriguingly, we observed that patients with baseline anti-Hu antibodies exhibited a higher incidence of immune-related toxicities of any type. This finding suggests that anti-Hu positivity may act as a surrogate marker of heightened cancer-driven immune activation, which could also underlie the development of non-neurological off-target toxicities (24). Moreover, we observed a trend toward a better survival outcome in patients with anti-Hu positivity. While the prognostic impact of baseline anti-Hu antibodies in SCLC outside the context of ICI therapy remains conflicting (24, 25), it has not yet been explored in patients undergoing ICI treatment. Further studies are warranted to elucidate the prognostic role of baseline anti-Hu antibodies in patients with SCLC treated with ICIs. Taken together, the increased incidence of irAEs and the trend toward better oncological outcome may reflect parallel manifestations of enhanced anti-tumor immune response (26).

Out study has several limitations. First, its small sample size, which hindered comparison between patients with and without neurological toxicities. Additionally, the relatively small number of patients with baseline IC-Abs prevented us from drawing definite conclusion regarding the risk of n-irAEs in this group. Second, we used commercial TBA and line-blot assay, which are known to have suboptimal sensitivity and specificity compared to in-house TBA and cell-based assay, respectively (14, 15, 27). Third, we classified patients with positive line-blot results but negative IIF-TBA as antibody-negative. This decision may have led to underestimation of true antibody positivity—particularly for SOX1 antibodies, which are not reliably detected by IIF-TBA (28). Forth, IC-Abs were not systematically retested after ICI therapy in patients with baseline positivity. This data might help to elucidate the impact of ICI therapy on subthreshold neuronal autoimmunity and should be investigated in future studies.

In conclusion, our findings suggest the presence of baseline IC-Abs in patients with SCLC is not necessarily associated with the occurrence of immune-related toxicities after ICI therapy. Although pre-treatment neural antibody positivity, particularly anti-Hu, should prompt closer monitoring for ir-AEs, it should not exclude patients from ICI therapy. Future large-scale prospective studies are needed to further elucidate the predictive and prognostic value of baseline neural antibodies in the setting of ICI treatment.

## Data Availability

The original contributions presented in the study are included in the article/**Supplementary Material**. Further inquiries can be directed to the corresponding author/s.

## References

[1] Horn L Mansfield AS Szczęsna A Havel L Krzakowski M Hochmair MJ . First-line atezolizumab plus chemotherapy in extensive-stage small-cell lung cancer. New Engl J Med. (2018) 379:2220–9. doi: 10.1056/NEJMoa1809064, PMID: 30280641

[2] Goldman JW Dvorkin M Chen Y Reinmuth N Hotta K Trukhin D . Durvalumab, with or without tremelimumab, plus platinum–etoposide versus platinum–etoposide alone in first-line treatment of extensive-stage small-cell lung cancer (CASPIAN): updated results from a randomised, controlled, open-label, phase 3 trial. Lancet Oncol. (2021) 22:51–65. doi: 10.1016/S1470-2045(20)30539-8, PMID: 33285097

[3] Postow MA Sidlow R Hellmann MD . Immune-related adverse events associated with immune checkpoint blockade. New Engl J Med. (2018) 378:158–68. doi: 10.1056/NEJMra1703481, PMID: 29320654

[4] Marini A Bernardini A Gigli GL Valente M Muñiz-Castrillo S Honnorat J . Neurologic adverse events of immune checkpoint inhibitors: A systematic review. Neurology. (2021) 96:754–66. doi: 10.1212/WNL.0000000000011795, PMID: 33653902

[5] Farina A Villagrán-García M Vogrig A Zekeridou A Muñiz-Castrillo S Velasco R . Neurological adverse events of immune checkpoint inhibitors and the development of paraneoplastic neurological syndromes. Lancet Neurol. (2024) 23:81–94. doi: 10.1016/S1474-4422(23)00369-1, PMID: 38101905

[6] Graus F Dalmau J . Paraneoplastic neurological syndromes in the era of immune-checkpoint inhibitors. Nat Rev Clin Oncol. (2019) 16:535–48. doi: 10.1038/s41571-019-0194-4, PMID: 30867573

[7] Graus F Vogrig A Muñiz-Castrillo S Antoine JCG Desestret V Dubey D . Updated diagnostic criteria for paraneoplastic neurologic syndromes. Neurol - Neuroimmunol Neuroinflammation. (2021) 8:e1014. doi: 10.1212/NXI.0000000000001014, PMID: 34006622 PMC8237398

[8] Vogrig A Fouret M Joubert B Picard G Rogemond V Pinto AL . Increased frequency of anti-Ma2 encephalitis associated with immune checkpoint inhibitors. Neurol Neuroimmunol Neuroinflamm. (2019) 6:e604. doi: 10.1212/NXI.0000000000000604, PMID: 31454760 PMC6705619

[9] Farina A Villagrán-García M Ciano-Petersen NL Vogrig A Muñiz-Castrillo S Taillandier L . Anti-hu antibodies in patients with neurologic side effects of immune checkpoint inhibitors. Neurol Neuroimmunol Neuroinflamm. (2023) 10:e200058. doi: 10.1212/NXI.0000000000200058, PMID: 36446613 PMC9709718

[10] Farina A Birzu C Elsensohn MH Picca A Muniz-Castrillo S Vogrig A . Neurological outcomes in immune checkpoint inhibitor-related neurotoxicity. Brain Commun. (2023) 5:fcad169. doi: 10.1093/braincomms/fcad169, PMID: 37389303 PMC10306160

[11] Fonseca E Cabrera-Maqueda JM Ruiz-García R Naranjo L Diaz-Pedroche C Velasco R . Neurological adverse events related to immune-checkpoint inhibitors in Spain: a retrospective cohort study. Lancet Neurol. (2023) 22:1150–9. doi: 10.1016/S1474-4422(23)00335-6, PMID: 37977714

[12] Farina A Villagrán-García M Vogrig A Joubert B . Central nervous system adverse events of immune checkpoint inhibitors. Curr Opin Neurol. (2024) 37:345–52. doi: 10.1097/WCO.0000000000001259, PMID: 38483130

[13] Guidon AC Burton LB Chwalisz BK Hillis JM Schaller T Reynolds KL . Consensus disease definitions for the spectrum of neurologic immune related adverse events. J Clin Oncol. (2021) 9:e002890. doi: 10.1200/JCO.2021.39.15_suppl.2647 PMC8291304 34281989

[14] Ruiz-García R Martínez-Hernández E Saiz A Dalmau J Graus F . The diagnostic value of onconeural antibodies depends on how they are tested. Front Immunol. (2020) 11. doi: 10.3389/fimmu.2020.01482, PMID: 32760403 PMC7372120

[15] Déchelotte B Muñiz-Castrillo S Joubert B Vogrig A Picard G Rogemond V . Diagnostic yield of commercial immunodots to diagnose paraneoplastic neurologic syndromes. Neurol Neuroimmunol Neuroinflamm. (2020) 7:e701. doi: 10.1212/NXI.0000000000000701, PMID: 32170044 PMC7136063

[16] Arriola E Wheater M Galea I Cross N Maishman T Hamid D . Outcome and biomarker analysis from a multicenter phase 2 study of ipilimumab in combination with carboplatin and etoposide as first-line therapy for extensive-stage SCLC. J Thorac Oncol. (2016) 11:1511–21. doi: 10.1016/j.jtho.2016.05.028, PMID: 27296105 PMC5063510

[17] Sato Y Fujiwara S Hata A Kida Y Masuda T Amimoto H . Clinical impact of pre-existing autoantibodies in patients with SCLC treated with immune checkpoint inhibitor: A multicenter prospective observational study. JTO Clin Res Rep. (2023) 4:100608. doi: 10.1016/j.jtocrr.2023.100608, PMID: 38162177 PMC10755358

[18] Vogrig A Muñiz-Castrillo S Desestret V Joubert B Honnorat J . Pathophysiology of paraneoplastic and autoimmune encephalitis: genes, infections, and checkpoint inhibitors. Ther Adv Neurol Disord. (2020) 13:1756286420932797. doi: 10.1177/1756286420932797, PMID: 32636932 PMC7318829

[19] Valencia-Sanchez C Sechi E Dubey D Flanagan EP McKeon A Pittock SJ . Immune checkpoint inhibitor-associated central nervous system autoimmunity. Eur J Neurol. (2023) 30:2418–29. doi: 10.1111/ene.15835, PMID: 37151179

[20] Vogrig A Dentoni M Florean I Cellante G Domenis R Iacono D . Prediction, prevention, and precision treatment of immune checkpoint inhibitor neurological toxicity using autoantibodies, cytokines, and microbiota. Front Immunol. (2025) 16. doi: 10.3389/fimmu.2025.1548897, PMID: 40181971 PMC11966491

[21] Verschuuren JJ Perquin M ten Velde G De Baets M Vriesman P v. B Twijnstra A . Anti-Hu antibody titre and brain metastases before and after treatment for small cell lung cancer. J Neurol Neurosurg Psychiatry. (1999) 67:353–7. doi: 10.1136/jnnp.67.3.353, PMID: 10449558 PMC1736513

[22] Chiu D Rhee J Gonzalez Castro LN . Diagnosis and treatment of paraneoplastic neurologic syndromes. Antibodies. (2023) 12:50. doi: 10.3390/antib12030050, PMID: 37606434 PMC10443237

[23] Farina A Villagrán-García M Fourier A Pinto AL Chorfa F Timestit N . Diagnostic and prognostic biomarkers in immune checkpoint inhibitor-related encephalitis: a retrospective cohort study. Lancet Regional Health - Europe. (2024), 44:44. doi: 10.1016/j.lanepe.2024.101011, PMID: 39170102 PMC11338149

[24] Graus F Dalmou J Reñé R Tora M Malats N Verschuuren JJ . Anti-Hu antibodies in patients with small-cell lung cancer: association with complete response to therapy and improved survival. J Clin Oncol. (1997) 15:2866–72. doi: 10.1200/JCO.1997.15.8.2866, PMID: 9256130

[25] Monstad SE Drivsholm L Storstein A Aarseth JH Haugen M Lang B . Hu and voltage-gated calcium channel (VGCC) antibodies related to the prognosis of small-cell lung cancer. J Clin Oncol. (2004) 22:795–800. doi: 10.1200/JCO.2004.01.028, PMID: 14990634

[26] Ricciuti B Naqash AR Naidoo J Sehgal K Miller A Kehl K . Association between immune-related adverse events and clinical outcomes to programmed cell death protein 1/programmed death-ligand 1 blockade in SCLC. JTO Clin Res Rep. (2020) 1:100074. doi: 10.1016/j.jtocrr.2020.100074, PMID: 34589955 PMC8474257

[27] Milano C Businaro P Papi C Arlettaz L Marmolejo L Naranjo L . Assessing commercial tissue-based assays for autoimmune neurologic disorders (I). Neurol Neuroimmunol Neuroinflamm. (2025) 12:e200410. doi: 10.1212/NXI.0000000000200410, PMID: 40393022 PMC12153943

[28] Arnaldos-Pérez C Vilaseca A Naranjo L Sabater L Dalmau J Ruiz-García R . Algorithm to improve the diagnosis of paraneoplastic neurological syndromes associated with SOX1 antibodies. Front Immunol. (2023) 14. doi: 10.3389/fimmu.2023.1173484, PMID: 37207233 PMC10191251

