# Supplementary figures and images for: Baseline neuronal antibodies in patients with small cell lung cancer are not necessarily associated with post-immune checkpoint inhibitors neurotoxicities
Source: Front Immunol. 2025 Nov 20;16:1681765. doi: 10.3389/fimmu.2025.1681765 (PMC12675432; doi:10.3389/fimmu.2025.1681765)

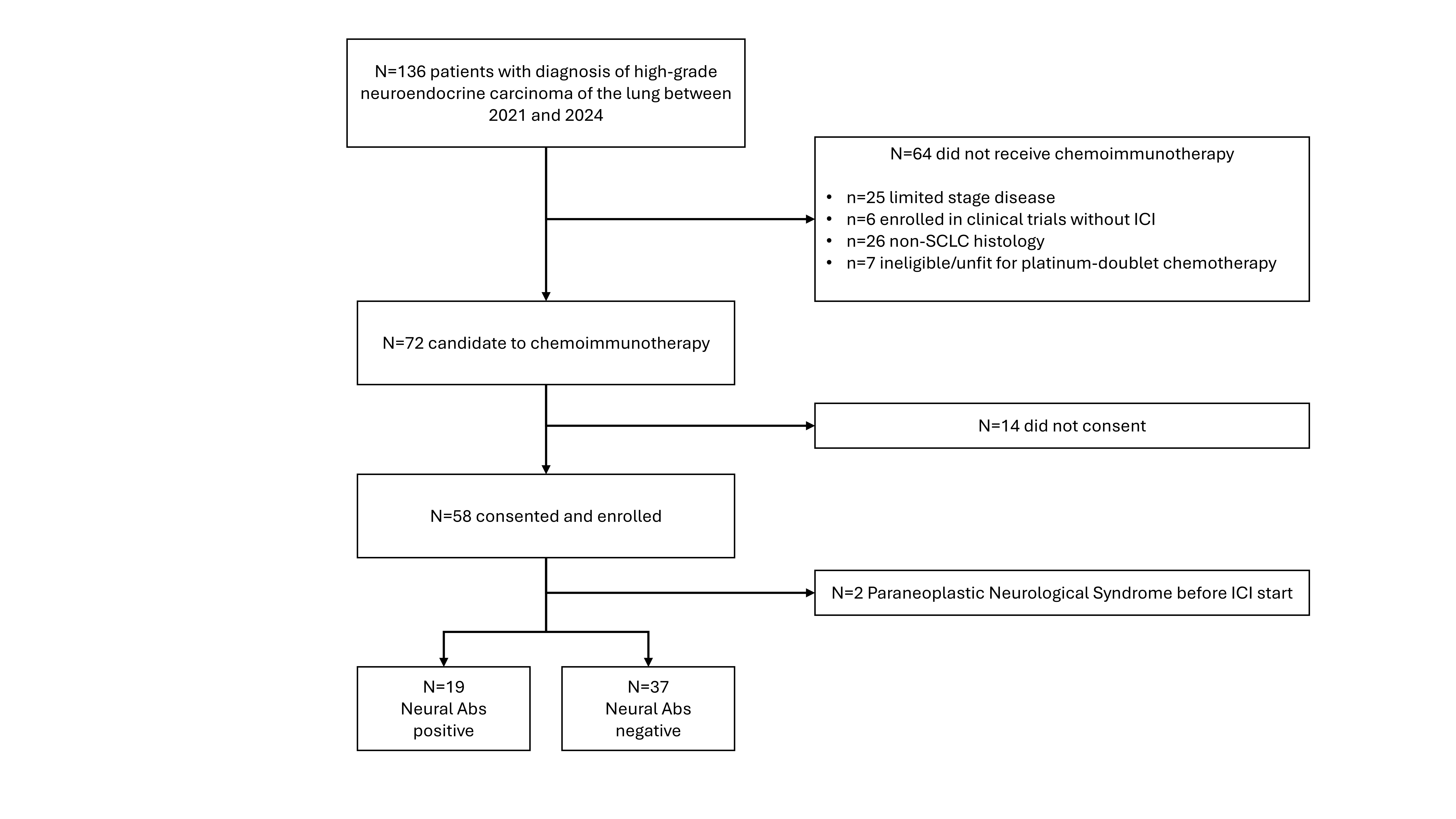

Supplement: Supplementary file 1 [file Image1.jpg]

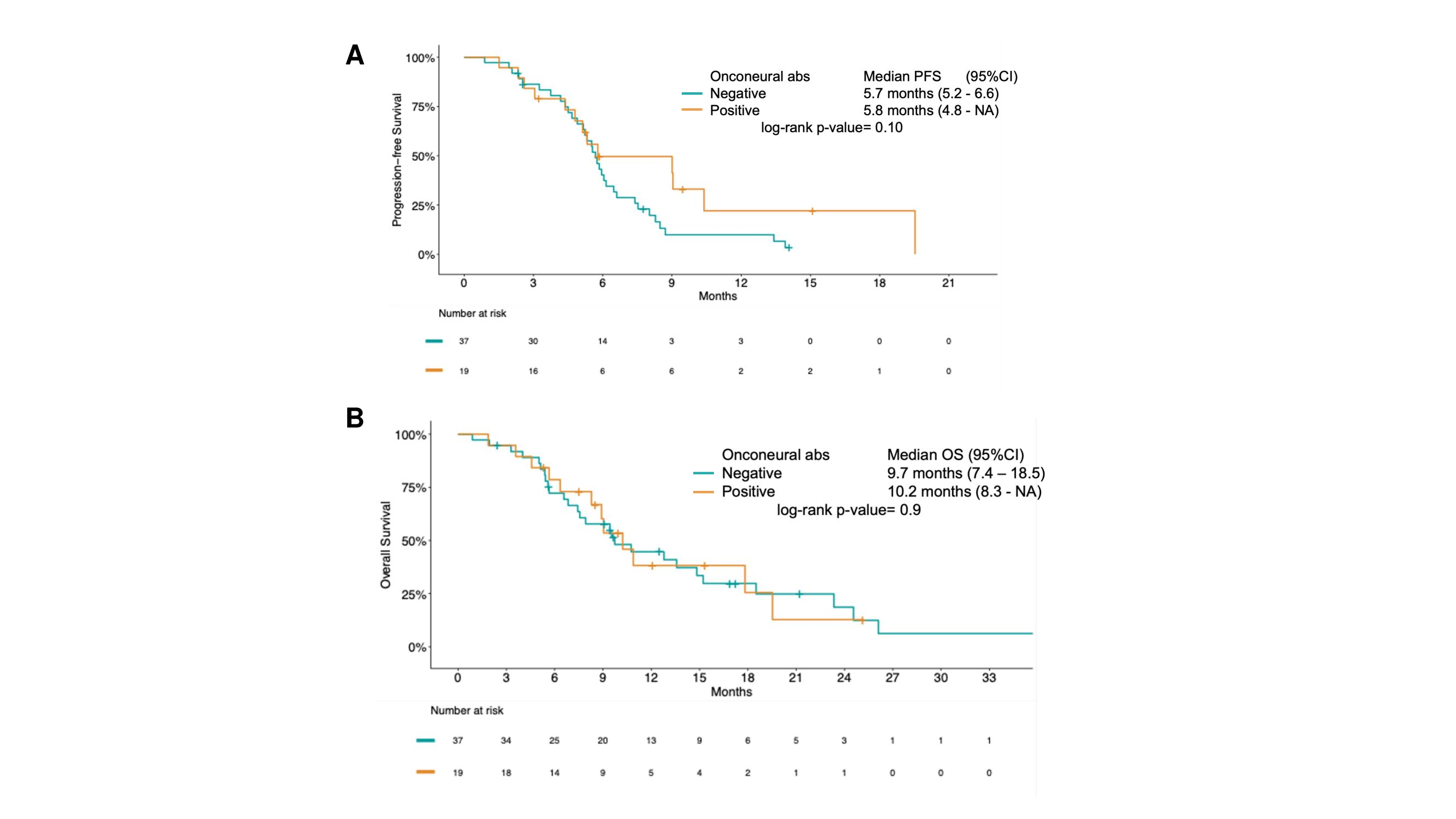

Supplement: Supplementary file 2 [file Image2.png]
