# Supplementary material for: Baseline neuronal antibodies in patients with small cell lung cancer are not necessarily associated with post-immune checkpoint inhibitors neurotoxicities
Source: Front Immunol. 2025 Nov 20;16:1681765. doi: 10.3389/fimmu.2025.1681765 (PMC12675432; doi:10.3389/fimmu.2025.1681765)
Supplement: Supplementary file 3 [file Table1.docx]

**Supplementary Table 1.** Patient characteristics by anti-Hu positivity.

|  | **Anti-Hu negative (N= 49)** | **Anti-Hu positive (N= 7)** | **p-value** |
| --- | --- | --- | --- |
| **Sex** |  |  |  |
| Female | 17 (34.7%) | 4 (57.1%) | 0.406 |
| Male | 32 (65.3%) | 3 (42.9%) |  |
| **Age** |  |  |  |
| Median [Min, Max] | 71.0 [51.0, 85.0] | 70.0 [55.0, 74.0] | 0.327 |
| **Pack-year** |  |  |  |
| Median [Min, Max] | 50.5 [0, 100] | 40.0 [33.0, 90.0] | 0.527 |
| **ECOG PS** |  |  |  |
| 0 | 15 (30.6%) | 2 (28.6%) | 1.00 |
| 1 | 34 (69.4%) | 5 (71.4%) |  |
| **Stage at diagnosis** |  |  |  |
| Extensive | 40 (81.6%) | 7 (100%) | 0.583 |
| Limited | 9 (18.4%) | 0 (0%) |  |
| **Ki-67** |  |  |  |
| Median [Min, Max] | 80.0 [50.0, 95.0] | 90.0 [70.0, 90.0] | 0.797 |
| **Metastatic sites** |  |  |  |
| Liver | 14 (28.6%) | 1 (14.3%) | 0.661 |
| Bone | 12 (24.5%) | 1 (14.3%) | 1.00 |
| Brain | 9 (18.4%) | 0 (0%) | 0.583 |
| **Type of ICI** |  |  |  |
| Atezolizumab | 34 (69.4%) | 4 (57.1%) | 0.372 |
| Durvalumab | 14 (28.6%) | 2 (28.6%) |  |
| Pembrolizumab | 1 (2.0%) | 1 (14.3%) |  |
| **ICI cycles** |  |  |  |
| Median [Min, Max] | 7.00 [1.00, 18.0] | 9.00 [4.00, 21.0] | 0.307 |
