# Supplementary material for: Baseline neuronal antibodies in patients with small cell lung cancer are not necessarily associated with post-immune checkpoint inhibitors neurotoxicities
Source: Front Immunol. 2025 Nov 20;16:1681765. doi: 10.3389/fimmu.2025.1681765 (PMC12675432; doi:10.3389/fimmu.2025.1681765)
Supplement: Supplementary file 4 [file Table2.docx]

| **Onconeural antibodies** | **irAEs** | | | | |
| --- | --- | --- | --- | --- | --- |
|  | Grade 3 | Grade 1-2 | *P* | No irAE | *P* |
| positive | 2 | 4 | 1.00 | 17 | 0.59 |
| negative | 2 | 6 |  | 35 |  |
| **Anti-Hu antibodies** |  | | | | |
| positive | 2 | 3 | 0.58 | 5 | 0.07 |
| negative | 2 | 7 |  | 47 |  |

**Supplementary Table 2**. Fisher’s test of association between grade 3 vs grade 1-2 immune-related adverse events (irAEs) by onconeural or anti-Hu antibody positivity, and between grade 3 irAEs occurence vs no by onconeural or anti-Hu antibody positivity.
