# Supplementary material for: Baseline neuronal antibodies in patients with small cell lung cancer are not necessarily associated with post-immune checkpoint inhibitors neurotoxicities
Source: Front Immunol. 2025 Nov 20;16:1681765. doi: 10.3389/fimmu.2025.1681765 (PMC12675432; doi:10.3389/fimmu.2025.1681765)
Supplement: Supplementary file 5 [file Table3.docx]

| **Antibody title** | **No irAE (N= 42)** | **irAE (N= 14)** | **p-value** |
| --- | --- | --- | --- |
| **ANA** |  |  |  |
| Negative | 22 (57.9%) | 7 (50.0%) | 0.755 |
| Positive | 16 (42.1%) | 7 (50.0%) |  |
| **ANA w/ borderline** |  |  |  |
| Negative | 17 (44.7%) | 3 (21.4%) | 0.200 |
| Positive | 21 (55.3%) | 11 (78.6%) |  |
| **Anti-ENA** |  |  |  |
| Negative | 11 (100%) | 1 (33.3%) | 0.033 |
| Positive | 0 (0%) | 2 (66.7%) |  |
| **Anti-Thyroid** |  |  |  |
| Negative | 36 (94.7%) | 10 (71.4%) | 0.038 |
| Positive | 2 (5.3%) | 4 (28.6%) |  |
| **ANCA** |  |  |  |
| Negative | 38 (100%) | 12 (85.7%) | 0.069 |
| Positive | 0 (0%) | 2 (14.3%) |  |

**Supplementary Table 3.** Antibody positivity by occurrence of immune-related adverse events (irAE). ANA: anti-nuclear antigen; ANA w/ borderline: borderline results (1:80) were assumed positive; ENA: extractable nuclear antigens, include anti-Sm, anti-RNP, anti-SS-A (Ro), and anti-SS-B (La), anti-Jo1, and anti Scl-70; anti-Thyroid includes thyroid stimulating hormone resecptor (TSH), thyroid perxidase (TPO), and thyreoglobulin (Tg); ANCA: anti-neutrophil cytoplasmic antibody, includes proteinase 3 (PR3) and myeloperoxidase (MPO).
