# Supplementary material for: Baseline neuronal antibodies in patients with small cell lung cancer are not necessarily associated with post-immune checkpoint inhibitors neurotoxicities
Source: Front Immunol. 2025 Nov 20;16:1681765. doi: 10.3389/fimmu.2025.1681765 (PMC12675432; doi:10.3389/fimmu.2025.1681765)
Supplement: Supplementary file 6 [file Table4.docx]

**Supplementary Table 4.** Multivariable linear regression model of the association of antibody positivity with irAEs. To avoid collinearity, only ANA w/borderline has been included in the model. ENA was not included in the model because all cases with available ENA (14/56, 25%) did not have detectable anti-Hu antibodies. ANA: anti-nuclear antigen; ANA w/ borderline: borderline results (1:80) were assumed positive; ENA: extractable nuclear antigens, include anti-Sm, anti-RNP, anti-SS-A (Ro), and anti-SS-B (La), anti-Jo1, and anti Scl-70; anti-Thyroid includes thyroid stimulating hormone resecptor (TSH), thyroid perxidase (TPO), and thyreoglobulin (Tg); ANCA: anti-neutrophil cytoplasmic antibody, includes proteinase 3 (PR3) and myeloperoxidase (MPO).

| **Covariate** | **OR** | **95%CI** | **p-value** |
| --- | --- | --- | --- |
| Anti-Hu positivity | 21.4 | 1.99-230.24 | 0.004 |
| ANA w/ borderline positivity | 1.52 | 0.28-8.17 | 0.623 |
| ANCA positivity | NE | 0-Infinite | 0.265 |
| Anti-thyroid positivity | 4.88 | 0.52-45.92 | 0.173 |
